# No evidence that footedness in pheasants influences cognitive performance in tasks assessing colour discrimination and spatial ability

**DOI:** 10.3758/s13420-019-00402-8

**Published:** 2020-01-08

**Authors:** Mark A. Whiteside, Mackenzie M. Bess, Elisa Frasnelli, Christine E. Beardsworth, Ellis J.G. Langley, Jayden O. van Horik, Joah R. Madden

**Affiliations:** 1 Centre for Research in Animal Behaviour, Psychology, University of Exeter, Exeter, EX4 4QG UK; 2 School of Life Sciences, University of Lincoln, Lincoln, LN6 7DL UK

**Keywords:** Associative learning, Laterality, Spatial learning, Footedness

## Abstract

The differential specialization of each side of the brain facilitates the parallel processing of information and has been documented in a wide range of animals. Animals that are more lateralized as indicated by consistent preferential limb use are commonly reported to exhibit superior cognitive ability as well as other behavioural advantages. We assayed the lateralization of 135 young pheasants (*Phasianus colchicus*), indicated by their footedness in a spontaneous stepping task, and related this measure to individual performance in either 3 assays of visual or spatial learning and memory. We found no evidence that pronounced footedness enhances cognitive ability in any of the tasks. We also found no evidence that an intermediate footedness relates to better cognitive performance. This lack of relationship is surprising because previous work revealed that pheasants have a slight population bias towards right footedness, and when released into the wild, individuals with higher degrees of footedness were more likely to die. One explanation for why extreme lateralization is constrained was that it led to poorer cognitive performance, or that optimal cognitive performance was associated with some intermediate level of lateralization. This stabilizing selection could explain the pattern of moderate lateralization that is seen in most non-human species that have been studied. However, we found no evidence in this study to support this explanation.

**Electronic supplementary material:**

The online version of this article (10.3758/s13420-019-00402-8) contains supplementary material, which is available to authorized users.

The functional specialization of either side of the brain (or nervous system in general) was previously uniquely attributed to humans, but in recent years lateralization has been shown to be widespread among vertebrates and invertebrates (Frasnelli, Vallortigara, & Rogers, 2012; Rogers, Vallortigara, & Andrew, 2013). In animals, lateralization of the brain is frequently revealed by behavioural lateralization, such as the limb preferentially used to perform actions (Bell & Niven, 2016; Found & St. Clair, 2017; Magat & Brown, 2009; McGrew & Marchant, 1999), the eye used to inspect the environment for feeding or checking for potential predators (Rogers, 2000; Rogers, Zucca, & Vallortigara, 2004), the side of body presented to peers (Jennings, 2012; Krakauer et al., 2016), or the direction of movement when catching prey (e.g., Kurvers et al., 2017), escaping from predators (e.g., De Santi, Sovrano, Bisazza, & Vallortigara, 2001), or coordinating with conspecifics (e.g., Frasnelli, Iakovlev, & Reznikova, 2012). In some cases, lateralization offers cognitive benefits to individuals: It spares neuronal tissue (Levy, 1969), preventing the simultaneous initiation of incompatible responses (Andrew, Mench, & Rainey, 1982; Cantalupo, Vila Pouca, & Brow, 1995), and facilitates separate and parallel processing in the two hemispheres (Rogers et al., 2004). In many cases stronger degrees of lateralization have been shown to relate to greater cognitive ability (for a review, see Rogers et al., 2013). Specifically, individuals with stronger degrees of lateralization had better memory in larval antlions (*Myrmeleon bore*; Miler, Kuszewska, & Woyciechowski, 2017) and *Drosophila* (Pascual, Huang, Neveu, & Préat, 2004); solving ability on visual discrimination tasks in parrots (Magat & Brown, 2009), chickens (*Gallus gallus*; Rogers et al., 2004), and pigeons (*Columba livia*; Güntürkün et al., 2000); motor skill in desert locusts (*Schistocerca gregaria*; Bell & Niven, 2016) and Australian parrots (Magat & Brown, 2009); and numerical skills in guppies (*Poecilia reticulata*; Dadda, Agrillo, Bisazza, & Brown, 2015). Such benefits of parallel processing and improved cognitive and motor abilities are suggested to be the forces driving the evolution of lateralization (Rogers, 2002). However, if lateralization confers so many advantages, it is still unclear why do we do not see animal populations that are uniformly and extremely lateralized, such as in humans (Corballis, 2012; McManus, 2002; Ströckens, Güntürkün, & Ocklenburg, 2013).

Variation in the extent of lateralization both within and between populations suggests that, at least in many nonhuman animals, there is a cost to lateralization. However, there are few studies that show such costs. Tropical poeciliids (*Brachyraphis episcopi*) that were more lateralized took longer to solve a maze task because they were fixated in turning in a particular (wrong) direction (Brown & Braithwaite, 2004). Strongly lateralized goldbelly topminnows (*Giraldinus falcatus*) performed poorly when discriminating between two shoals of differing sizes compared with less strongly lateralized individuals (Dadda, Zandona, Agrillo, & Bisazza, 2009). In humans, it has been suggested that there are nonlinear benefits of lateralization on cognitive performance—for instance, in a word-matching game, a moderately asymmetrical brain offered the best cognitive outcomes (Hirnstein, Leask, Rose, & Hausmann, 2010). However, in many of these studies, there is often a single cognitive ability being assessed, and the relationship between lateralization and performance has been demonstrated to be task dependent (Boles, Barth, & Merrill, 2008). Therefore, a degree of laterality that may benefit performance on one task may not benefit performance in another—hence, it is important to understand the costs and benefits of laterality within a single population across multiple cognitive tasks and domains (Dadda et al., 2015). Difficulty in assessing large numbers of animals makes it challenging to test for linear and nonlinear influences of lateralization on cognitive performance, particularly when wanting to compare the relationship with laterality for multiple tasks across cognitive domains.

Pheasants (*Phasianus colchicus*) offer an unusual system to help understand the costs of lateralization with respect to a broad set of cognitive abilities. One-day-old chicks can be reared in controlled environments where we can train large numbers of individuals to enter testing chambers to assess cognitive performances on a series of colour discrimination tasks (van Horik, Langley, Whiteside, & Madden, 2018) and on tasks to assess spatial ability (Langley, van Horik, Whiteside, Beardsworth, & Madden, 2018; Whiteside, Sage, & Madden, 2016). Chicks are trained to voluntarily enter the testing chamber, which allows the pheasants to complete trials in isolation without the fear of being caught, coerced, or stressed, which can influence cognitive performance (de Kloet, Oitzl, & Joëls, 1999; Mendl, 1999). Testing in isolation removes competition that may distort cognitive performance (Mendl, 1999). Testing in isolation also removes opportunities for social learning. Individual pheasants did not show consistent performance across a broad suite of cognitive tasks, suggesting that they did not exhibit domain-general intelligence (‘g’; van Horik, Langley, Whiteside, Laker, & Madden, 2018). Therefore, laterality may benefit one task more than another. Critically, for studies of lateralization, pheasants are Galliformes, like chickens, a well-studied model system for laterality in birds (e.g., Rogers et al., 2004; Tommasi & Vallortigara, 1999); they have lateral placed eyes; they eat both live prey and mixed grain (Hill & Robertson, 1988); and they live in groups (Ridley & Hill, 1987; Whiteside, van Horik, Langley, Beardsworth, Capstick, & Madden, 2018). These factors suggest that an asymmetrical brain could offer survival advantages: detecting predators; selecting nutritious food items; and coordinating group actions, respectively. However, we do not observe extreme lateralization (manifested by preferential footedness) at the population level in pheasants. Although some individuals do have a marked preference to use one foot over the other, the overall population showed only a slight right-footed bias (Whiteside, Bess, Frasnelli, Beardsworth, Langley, van Horik, & Madden, 2018), similar to the domestic chicken (Tommasi & Vallortigara, 1999), Japanese quail (*Coturnic cortunix japonica*) and bobwhite quail (*Colinus virginianus*; Casey, 2005). This constraint on extreme levels of footedness may be explained by the observation that after release into the wild, pheasants that had greater degrees of footedness were the ones with lower probability of survival (Whiteside et al., 2018). Survival may also be influenced by performance in cognitive tasks (Madden, Langley, Whiteside, Beardsworth, & van Horik, 2018). Therefore, one mechanism constraining the exaggeration of lateralization in pheasants, as indicated by preferential limb use, could be if extreme footedness leads to poor cognitive performance, which then inhibits survival. Therefore, we wanted to test whether an individual’s footedness was related to their performance in a suite of cognitive tasks.

Lateralization of the brain may be assessed by morphological measures of brain (region) tissue or differential patterns of neural activity across hemispheres (Ocklenburg & Güntürkün 2017; Rogers et al., 2013). However, these methods may be highly invasive or terminal such that making links to cognitive performance is difficult or impossible. Instead, brain lateralization may manifest in behavioural side biases (Ocklenburg & Güntürkün 2017; Rogers et al., 2013) that can be observed in free-living individuals. Unfortunately, laterality is not a unitary trait, but instead, different areas of the brain and different functional specialization might be independently lateralized or not be lateralized at all, and this is shown by the fact that different tasks testing for lateralization proxies barely correlate with each other (Ocklenburg & Güntürkün, 2017). We used an assay of preferential limb (foot) use to indicate brain lateralization—specifically, by monitoring which foot that a pheasant used to step up onto, or to step over, a barrier (see Whiteside et al., 2018). Such measures of limb preference, or more generically, differences in the use of limbs, are commonly used as indicators of lateralization in many vertebrate species, as it indicates a cerebral dominance of the controlateral hemisphere (Versace & Vallortigara, 2015). Preferential foot use has previously been used to assess lateralization in the closely related species (Casey, 2005). We then assessed the cognitive performance of individuals using a suite of tasks that assess several different cognitive domains. We chose these tasks because in other species, performance has been shown to be influenced by lateralization, although this has not been related to footedness. Using conditioning procedures, it has been shown that the right eye/left hemisphere is dominant in tasks involving colour, shape, and object discriminations in pigeons (Diekamp, Prior, & Güntürkün, 1999; also see Vallortigara, 2004). For tasks testing spatial ability and navigation, in domestic chicks the right hemisphere has been shown to govern large-scale geometry of the environment, and both hemispheres play a role in local, nongeometric cues (Vallortigara, Pagni, & Sovrano, 2004). Rats also show hemispheric differences in spatial cognition similar to those observed in chicks (LaMendola & Bever, 1997). Because of the logistic demands of conducting cognitive testing, we split out study population in two, and presented half of the birds with a series of binary colour discrimination tasks, whereby they had to learn which colour was rewarded (three separate tests); the other half of birds were presented with a series of tasks to assess their spatial ability, where the birds were required to locate rewards in a series of arrays (three separate tests). We assayed an individual’s cognitive performance and determined whether this was related to their strength of footedness. We made two predictions. Firstly, if exaggerated footedness in pheasants leads to increased mortality because it retards cognitive performance, then we expect a significant negative linear relationship between footedness and cognitive performance. Secondly, if, like in humans and as suggested by Whiteside and colleagues (2018), there is a benefit of a mildly asymmetrical brain, then we expect a quadratic relationship between footedness and cognitive performance, such that individuals with intermediate degrees of footedness perform best on some or all cognitive tasks.

## METHOD

### The rearing system

For 10 weeks, we reared 259 individually marked pheasants from 1 day old and placed them into one of four identical aviaries at North Wyke Farm, Devon, UK. For the first 2 weeks of life, chicks had access to a heated house (2 m × 2 m). From 2 weeks they had access to an outdoor shelter (2 m × 4 m). At 3 weeks old they had further access to an outdoor enclosure (4 m × 12 m). Within the rearing house, and separated by a sliding door, was a testing chamber (0.75 m × 0.75 m) that exited into the outdoor shelter. We provided food and water ad lib and in excess and opportunities for perching. Sex was determined by morphological traits such as plumage colouration and presence of wattles and spurs.

### Measuring footedness

The pheasants used in this experiment were the same population that were assessed for footedness in Whiteside et al. (2018). While shaping the chicks to enter the testing chamber (cee Cognitive Training, Apparatus, and Testing), we assessed their footedness. We placed a block (25 cm × 5 cm × 5 cm) in front of the exit door (that was placed centrally and directly opposite any testing apparatus in order to not introduce possible position biases) and recorded the foot that each bird used to step onto/over the block as they exited the chamber. We only considered instances when birds were stationary prior to stepping in order to remove the effect of gait on foot choice. We only used data collected after the pheasants were older than 16 days because, in chickens, prior to this age, the chicks showed no consistency in footedness (Dharmaretnam, Vijitha, Priyadharshini, Jashini, & Vathany, 2002). Footedness was measured by calculating their laterality index—that is, an index score defined by the formula (left − right)/(left + right), where left and right are the instances that each individual used the left or the right foot, respectively. From this index, we constructed a degree of footedness by taking the absolute value of the laterality index, regardless of direction. A degree of footedness of zero corresponded to individuals with no foot preference, whereas a degree of one corresponded to individuals who exclusively used either their left or their right foot. We calculated the degree of footedness only for birds that were observed stepping up on to the block at least four times, and any bird for which we had fewer observations were excluded from further analysis.

### Cognitive training, apparatus, and testing

We habituated all birds from hatching to enter the testing chamber of their own volition. Within the testing chamber, we shaped the birds to approach a poke box (120 mm × 400 mm) containing 10 wells in a 5 × 2 design (diameter = 20 mm, depth = 15 mm), and then to peck through a layer of crepe paper and retrieve mealworms concealed in each well. At the age of 3 weeks, the birds were assigned to one of two treatment groups as part of another study. Two of the replicated pens were assigned to a treatment where they were presented with three colour discrimination tasks (see Colour Discrimination Tasks for the protocols). The second pair of replicates were assigned to a treatment where they were presented with three tasks designed to test spatial ability (see Spatial Tasks for the protocols).

For each cognitive task, at the beginning of a session, an individual would voluntarily enter the testing chamber from its house where it was then presented with the test apparatus. A mealworm was placed in the middle of the poke box surface to centralize the bird prior to their first trial of each session. The protocol for each task is detailed separately below. Upon completion of a designated number of trials (10 for binary discrimination task, four for 10-well task), the session was over and the bird would then exit the chamber into the outdoor enclosure. If the birds did not interact with the apparatus within 2 minutes or if they showed signs of stress, testing was ceased, and then they were allowed to leave the testing arena.

#### Colour discrimination tasks

Birds assigned to the colour discrimination treatment were presented with three separate binary discrimination tasks. In each task, the bird was required to discriminate between two wells (diameter = 20 mm, depth = 15 mm), the contents of which were concealed by crepe paper that differed only in the colour around its border. One of the two colours was consistently rewarded and the other was unrewarded. If the bird pecked at the rewarded well, it was scored as a correct choice, the bird was allowed to eat the mealworm, and the poke box was removed. If the bird pecked at the unrewarded well, the poke box was promptly removed and we scored an incorrect choice. Another trial was then presented until the individual had completed 10 trials in the session. The spatial location of each coloured well was pseudo-randomized, with no more than three rewarded wells being in the same location consecutively for each five pairs that were presented. There were five sessions over a 3-day period (one session conducted in the morning and one session conducted in the afternoon), totalling 50 trials for each pair. The birds were first presented with the blue–green colour combination in which the blue well was rewarded. We then presented the birds with the yellow–pink colour combination in which the yellow well was rewarded. We finally presented the birds with the dark-green–orange colour combination where the dark-green well was rewarded.

#### Spatial tasks

##### Binary choice: Left–right

At the age of 3 weeks, the birds were presented with the same poke box used in the colour discrimination tasks, but this time there was no colour bordering the wells. We presented a binary choice between two wells that were arranged horizontally (left–right) and separated by 1.2 cm and covered with crepe paper. The left well was consistently rewarded, and if pecked, we recorded a correct choice and the bird was allowed to consume the mealworms before the box was removed. The right well was unrewarded, and if pecked, we recorded an incorrect choice and the box was immediately removed. Like the colour discrimination tasks, each bird completed 50 trials presented in five sessions each of 10 binary choice trials over 3 days.

##### Binary choice: Top–bottom

Upon completion of the left–right tasks, at the age of three and a half weeks, the birds were presented with a top-bottom task. This matched the left–right task described above, but the two wells were arranged vertically (1.2 cm apart). The furthest well from the bird was consistently rewarded, and the closest well was not rewarded. Again, the birds had five sessions of 10 binary choices over a 3-day period totalling 50 trials.

##### 10-well spatial task

When the birds were 4 weeks old, we presented them with the same 10 well poke box that was used during the habituation and training process. However, unlike in training, where every well was rewarded, we rewarded only a single well (positioned on the row farthest away from the bird and fourth from the left), while the other nine were empty and all were covered with crepe paper. We recorded the number of errors, defined as pecking an unrewarded well, made prior to choosing the rewarded well. We ignored repeated visits to incorrect wells and only considered the first incorrect choices. Each bird completed 18 trials presented in four sessions each of four trials, and a final session of two trials.

### Calculating cognitive performance measures

We calculated how well the individual learned each task by generating learning curves using generalized linear models (GLM). For all binary choice tasks (all colour discrimination tasks and the left–right and top–bottom spatial tasks) we included the performance for each trial (correct/incorrect) as the response variable, with trial number as a fixed factor. Using a binomial error structure, we calculated the predicted probability that an individual would choose correctly on the first (X_first_) and final trials (X_final_); derived from the equation: *Y* = 1/(1 +  *exp* [−(*b*0 + *b*1*X*]), where b_0_ represents the intercept and b_1_ represents the slope estimate. A bird with a high X_final_ on the binary choice tasks had a higher probability of choosing correctly on the final trial and therefore a ‘better’ cognitive ability than birds with a low X_final_.

For the multiple-choice 10-well spatial task, we generated a learning curve for each bird, with the number of errors made on each trial as the response variable and trial as a fixed factor. We used a Poisson error structure and calculated the predicted number of errors an individual would make on the on the first (X_first_) and on their final trial (X_final_) using the formula: *Y* =  *exp* (*b*0 + (*b*1*X*)). A bird with a high X_final_ on the multiple 10-well spatial task was predicted to make more errors on the final trial than would a bird with a lower X_final_, and therefore would be considered to have ‘poorer’ cognitive ability. Learning curves were generated using R Version 3.4.1 (R Development Core Team, 2014).

### Statistical analysis

We observed 135 pheasants (66 colour discrimination tasks; 69 spatial discrimination tasks) stepping onto or over a block more than four times, and therefore they could be used in the subsequent statistical analyses.

We used a repeated-measures ANOVA to assess whether individuals improved between their X_first_ and X_final_ cognitive performance scores for each task separately. In the model we included individual ID as a repeated measure and sex as a fixed factor.

To determine whether degree of footedness predicted cognitive performance, we constructed two separate models for each task. The first model was a simple GLM assessing whether cognitive performance (X_final_) could be predicted by the strength of an individual’s footedness (footedness index as a covariate), the sex of the individual (Female:Male) and the direction of the footedness (Left:None:Right) and the interaction between an individual’s sex and their strength of footedness, and the interaction between the strength and direction of footedness of an individual.

In the second model, we separated birds into eight ordinal categories depending on their strength of footedness (0 < X ≤ 0.1; 0.1 < X ≤ 0.2; 0.2 < X ≤ 0.3; 0.3 < X ≤ 0.4; 0.4 < X ≤ 0.5; 0.5 < X ≤ 0.6; 0.6 < X ≤ 0.7; and X > 0.7). We then asked whether cognitive performance was influenced by the categorical strength of footedness, sex, the individual’s direction of footedness, and the interaction between strength of footedness and sex and the interaction between the strength and direction of footedness. We then conducted a quadratic polynomial contrast to determine whether there was a quadratic relationship between strength of footedness and cognitive performance. Therefore, we ran a total of 12 models. This exposes us to the risk of committing a Type II error because of multiple testing. We corrected for multiple testing by adjusting our alpha value to 0.004 (0.5/12). Repeated-measures ANOVAs and GLMs were conducted in SPSS v25.

## ETHICAL CONSIDERATION

All work was conducted under UK Home Office Licence PPL 30/3204. Pheasants were habituated to human contact from 1 day old and were shaped to enter testing chambers and approach apparatus using mealworm rewards. Birds could choose whether to participate in the task. Birds that exhibited signs of stress (e.g., pacing) were permitted to leave the testing arena, and their lack of participation was recorded. All husbandry adhered to the Code of Practice for the Welfare of Gamebirds Reared for Sporting Purposes (Department for Environment, Food and Rural Affairs, 2009).

## RESULTS

**Fig. 1:**
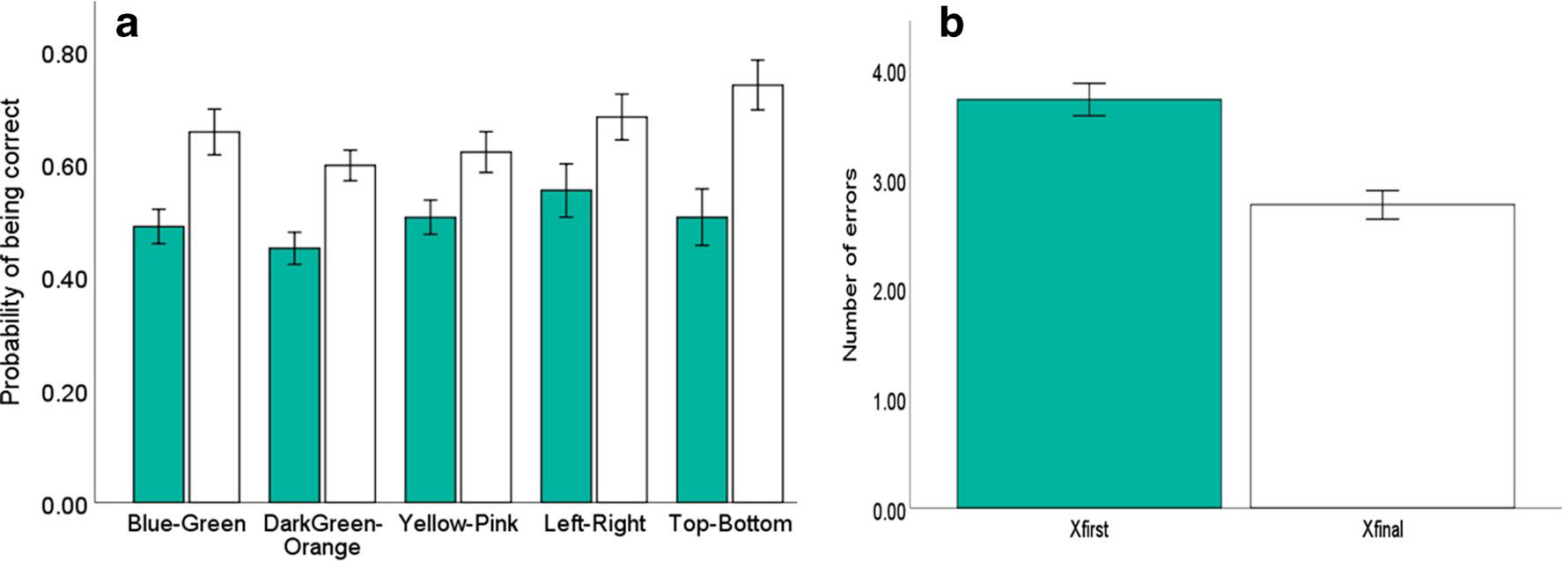
**a** The mean predicted probability of being correct on the first trial (green bars) and the mean predicted probability of being correct on the final trial (white bars) for each colour discrimination task and the two binary tasks assessing spatial ability. **b** The mean predicted number of errors made on the first trial and the final trial for pheasants presented with the 10-well tasks assessing spatial ability. Error bars ±1 *SE*

Pheasants showed evidence of learning in all the cognitive tasks that we presented, having a higher predicted probability of choosing correctly in their final trial compared with their first trial for all binary colour discrimination and spatial discrimination tasks (see Table 1 and Fig. 1a) and had a lower number of predicted errors in their final trial compared with their first trial in the 10-well spatial task (see Table 1 and Fig. 1b). Only on a single task (yellow–pink) did the sex of the bird influence an individual’s cognitive performance, with males having a higher predicted probability of being correct on the final trial compared with females (see Table 1).

**Table 1:** The mean performance and standard deviation for the first trial and last trial for all tasks

Task	First: $\overline{\mathrm{x}}$ (*SD*)	Final: $\overline{\mathrm{x}}$ (*SD*)	Variable	*df*	*df*(error)	*F*	*p*
Blue–green	**0.49 (0.12)**	**0.66 (0.17)**	**Improvement**	**1**	**64**	**24.759**	**<.001**
			Improvement*Sex	1	64	0.489	.49
			Sex	1	64	3.42	.07
Yellow–pink	**(0.51 (0.12)**	**0.62 (0.15)**	**Improvement**	**1**	**64**	**16.344**	**<.001**
			Improvement*Sex	**1**	**64**	**4.201**	**.045**
			Sex	1	64	0.377	.54
Dark-green–orange	**0.58 (0.11)**	**0.59 (0.09)**	**Improvement**	**1**	**64**	**34.555**	**>.001**
			Improvement*Sex	**1**	**64**	**6.063**	**.02**
			**Sex**	**1**	**64**	**9.005**	**.004**
Left–right	**0.55 (0.20)**	**0.68 (0.17)**	**Improvement**	**1**	**67**	**11.864**	**.001**
			Improvement*Sex	1	67	0.011	.92
			Sex	1	67	0.041	.84
Top–bottom	**0.51 (0.21)**	**0.74 (0.18)**	**Improvement**	**1**	**67**	**54.582**	**<.001**
			Improvement*Sex	1	67	3.48	.06
			Sex	1	67	0.7	.8
10-well	**3.73 (1.23)**	**2.76 (1.07)**	**Improvement**	**1**	**67**	**18.102**	**<.001**
			Improvement*Sex	1	67	2.139	.15
			Sex	1	67	0.095	.76

*Note.* For the binary tasks (every task but the 10-well task), the performance is measured as the predicted probability of being correct. For the 10-well task, the performance is the predicted number of errors made. Output of a repeated-measures ANOVA for each task presented to the pheasants. Improvement in each task is the relationship between the performance on the first trial and the performance on the final trial

**Fig. 2:**
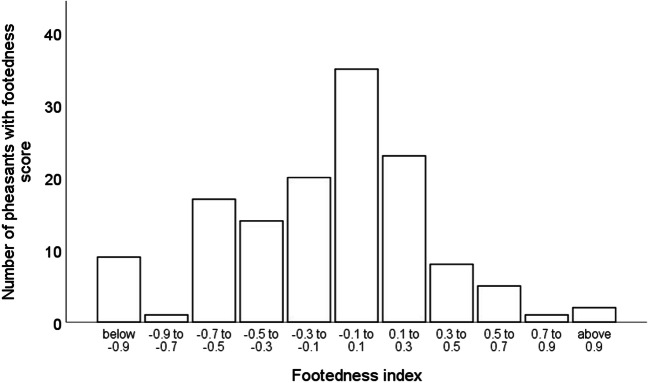
The strength and direction of footedness, measured as a footedness index based on the number of times an individual was observed using their left or right foot to step onto/over an obstacle—formula: (left − right)/(left + right)—for all pheasants that were either presented with colour and spatial discrimination tasks. Negative numbers represent a right bias, with −1 representing an individual that has a fully right footed bias. Positive numbers represent a left bias, with +1 representing an individual that has a fully left footed bias. An individual with zero footedness index has no bias.

**Fig. 3:**
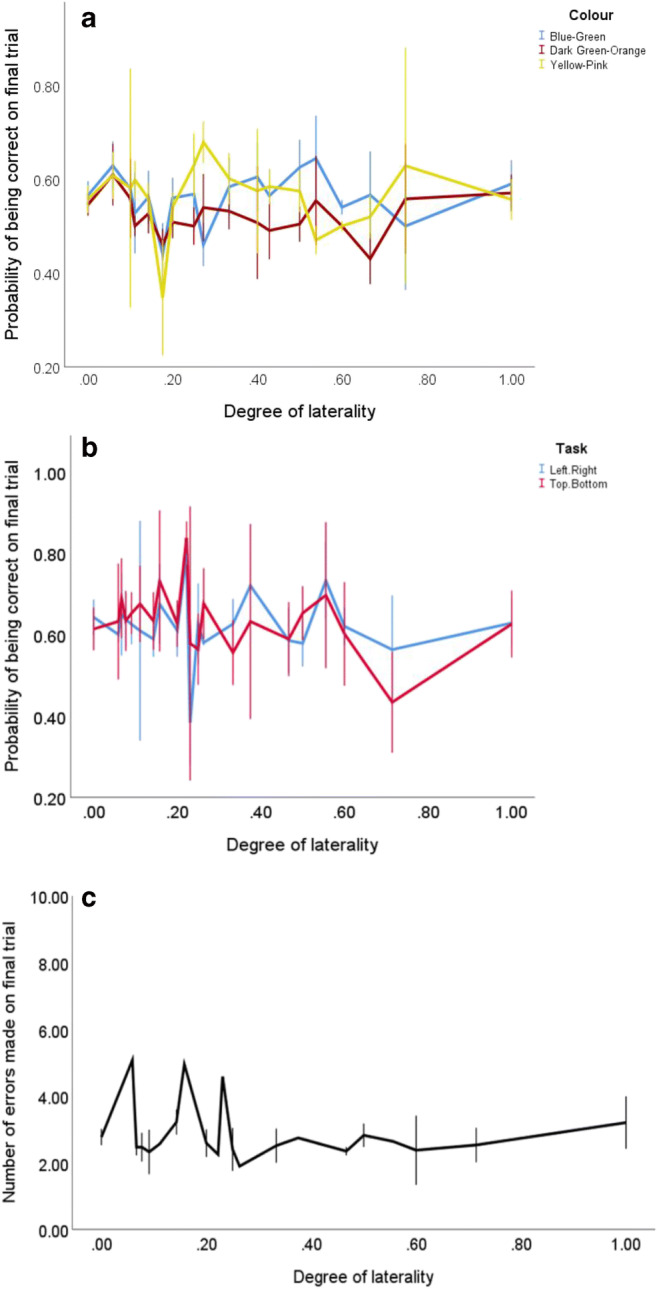
The relationship between an individual’s strength of footedness and (**a**) the predicted probability of being correct on the final trial for all colour discrimination tasks; (**b**) the predicted probability of being correct on the final trial for both binary tasks used to assess spatial ability; (**c**) the predicted number of errors made on the final trial of the 10-well tasks used to assess spatial ability. Error bars ±1 *SE*

Pheasants exhibited clear individual differences in their strength of footedness (see Fig. 2), which did not relate to their cognitive performance (X_final_) in any of the colour discrimination tasks (see Table 2 and Fig. 3a), either of the binary spatial tasks (Table 3, Fig. 3b) or the 10-well spatial task (Table 3, Fig. 3c), after adjusting the α to account for multiple testing. There were no significant interactions between the strength of an individual’s footedness and sex, or their direction of footedness on their performance on any of the tasks (see Table 2) after adjusting the alpha to account for multiple testing. There were also no quadratic relationships between an individual’s footedness and cognitive performance (X_final_) on any of the tasks, again, after adjusting the α to account for multiple testing.

**Table 2:** The output of the two GLMs performed on the data for all colour discrimination tasks

Task	Model	Variable	*df*	*df*(error)	*F*	*p*
Blue–green	i	Strength (Covariate)	1	59	7.826	0.01
		Sex	1	59	2.29	0.14
		Direction	2	59	3.29	0.04
		Strength × Sex	1	59	0.60	0.44
		Strength × Direction	1	59	5.63	0.02
	ii	Strength (Categorical)	7	46	3.29	0.06
		Sex	1	46	0.96	0.33
		Direction	2	46	0.38	0.68
		Strength × Sex	4	46	3.20	0.02
		Strength × Direction	5	46	1.70	0.16
		Strength (Quadratic)	contrast estimate:	−0.16	0.15	
Yellow–pink	i	Strength (Covariate)	1	59	0.71	0.40
		Sex	1	59	4.27	0.04
		Direction	2	59	0.24	0.79
		Strength × Sex	1	59	1.88	0.18
		Strength × Direction	1	59	0.85	0.36
	ii	Strength (Categorical)	7	46	0.50	0.83
		Sex	1	46	0.70	0.41
		Direction	2	46	0.29	0.75
		Strength × Sex	4	46	0.26	0.90
		Strength × Direction	5	46	0.30	0.91
		Strength (Quadratic)	contrast estimate:	−0.04	0.76	
Dark-green–orange	i	Strength (Covariate)	1	59	0.57	0.45
		Sex	1	59	0.04	0.85
		Direction	2	59	1.04	0.36
		Strength × Sex	1	59	0.96	0.33
		Strength ×Direction	1	59	0.38	0.54
	ii	Strength (Categorical)	7	46	0.72	0.66
		Sex	1	46	0.85	0.36
		Direction	2	46	0.06	0.94
		Strength × Sex	4	46	0.14	0.97
		Strength × Direction	5	46	0.83	0.53
		Strength (Quadratic)	contrast estimate:	−0.03	0.71	

*Note.* Assessing how cognitive performance (predicted probability of being correct on the final trial) is influenced by (i) the strength of footedness, the sex of the bird (Female:Male), the direction of the laterality (Left:None:Right), and the interaction between the strength of footedness and the sex of the bird and the interaction between the strength of footedness and the direction; (ii) the same factors as in Model I, but with strength of footedness as a categorical factor to allow for strength of footedness to be assessed as a quadratic function

**Table 3:** The output of the two GLMs performed on the data for all tasks

Task	Model	Variable	*df*	*df*(error)	*F*	*p*
Left–right	i	Strength (Covariate)	1	62	042	.52
		Sex	1	62	0.10	.73
		Direction	2	62	1.28	.28
		Strength × Sex	1	62	0.26	.61
		Strength × Direction	1	62	0.82	.37
	ii	Strength (Categorical)	7	47	0.57	.78
		Sex	1	47	0.36	.55
		Direction	2	47	1.83	.17
		Strength × Sex	5	47	1.85	.12
		Strength × Direction	5	47	1.50	.21
		Strength (Quadratic)	contrast estimate:		−0.05	0.06
Top–bottom	i	Strength (Covariate)	1	62	0.26	.62
		Sex	1	62	1.09	.30
		Direction	2	62	0.11	.90
		Strength × Sex	1	62	0.02	.89
		Strength × Direction	1	62	0.88	.35
	ii	Strength (Categorical)	7	47	0.35	.93
		Sex	1	47	2.22	.14
		Direction	2	47	0.75	.93
		Strength × Sex	5	47	0.51	.76
		Strength × Direction	5	47	0.85	.52
		Strength (Quadratic)	contrast estimate:		−0.05	.09
10-well	i	Strength (Covariate)	1	62	0.14	.71
		Sex	1	62	0.24	.63
		Direction	2	62	0.33	.72
		Strength × Sex	1	62	0.05	.83
		Strength × Direction	1	62	1.37	.25
	ii	Strength (Categorical)	7	47	0.19	.99
		Sex	1	47	2.47	.12
		Direction	2	47	0.06	.94
		Strength × Sex	5	47	1.05	.40
		Strength × Direction	5	47	0.78	.57
		Strength (Quadratic)	contrast estimate:		0.34	.51

*Note.* Assessing spatial ability assessing how cognitive performance is influenced by (i) the strength of footedness, the sex of the bird (Female:Male), the direction of the laterality (Left:None:Right), and the interaction between the strength of footedness and the sex of the bird and the interaction between the strength of footedness and the direction; (ii) the same factors as Model i, but with strength of footedness as a categorical factor to allow for strength of footedness to be assessed as a quadratic function. Cognitive performance on the two binary tasks was the predicted probability of choosing correctly on the final trial. Cognitive performance on the 10-well tasks was the predicted number of errors made on the final trial

## DISCUSSION

Pheasants exhibited individual differences in the strength of footedness expressed in a step-up task, previously suggested to indicate brain lateralization (Casey, 2005; Ocklenburg & Güntürkün, 2017; Rogers et al., 2013). However, we found no evidence that the strength of footedness was related to the cognitive performance of pheasants on a series of colour discrimination or spatial ability tasks. Therefore, we cannot provide support for the notion that enhanced footedness improves or inhibits cognitive ability in pheasants. Few studies have reported a lack of relationship between cognitive performance and lateralization, assessed either behaviourally or through brain (region) differences. Instead, the majority of published studies show a positive relationship between lateralization and cognitive performance ability across a wide range of tasks and taxa (for a review, see Rogers et al., 2013), and only a few published studies show a negative relationship (Brown & Braithwaite, 2004; Dadda et al., 2009). We also found no support for the hypothesis that a midlevel strength of footedness provided the strongest prediction of cognitive ability as has been suggested for humans (Hirnstein et al., 2010). In nonhuman animals, this quadratic relationship has not been detected, probably due to the requirement of testing a large number of animals.

Why do we not see the positive relationship between extent of footedness and cognitive performance that has been shown or suggested in many other systems? We suggest four possible explanations. Firstly, we may not be testing cognitive performances that could ever benefit from brain lateralization. However, we feel that this is not the case, given both the previous reports of a positive relationship between cognitive performance and lateralization (Dadda et al., 2015; Güntürkün et al., 2000; Magat & Brown, 2009; Rogers et al., 2004) and our understanding of neural architecture and the arrangement of brain regions and their interconnections (for a review, see Rogers et al., 2013). In other species, there is clear differential hemispheric relationships with cognition particularly in the avian hippocampus (Tommasi, Gagliardo, Andrew, & Vallortigara, 2003). Secondly, our measure of lateralization, footedness, may not be relevant for the tasks we provided. Lateralization can express itself as the asymmetrical use of paired body organs/limbs (Versace & Vallortigara, 2015). Depending on the specific task, different parts of the body are used. Thus, we may expect an asymmetrical use of limbs on tasks involving the use of limbs, and an asymmetrical use of eyes in tasks that are guided visually. Footedness may not directly relate to the performance of the cognitive tasks we presented to the pheasants because these tasks did not depend on foot use, but rather on visual perception of colour or spatial cues and accessing the reward using the bill. Thus, it is possible that our animals, when tested in a colour discrimination task, had a strong preference for a specific eye in visually guiding their behaviour rather than a preference for the foot that they stood on. Eye preference has been used as a proxy of lateralization in tasks involving visual discrimination. However, we did not test birds for eye preference as well as footedness, so we cannot test this explanation. Previous studies explicitly measured limb preference during the performance of the task deemed to be cognitively demanding—for example, handedness in chimps (*Pan troglodytes*) during foraging behaviour using a stick to fish termites (McGrew & Marchant, 1999). In our study, we attempted to link the measure of lateralization assessed in a behaviour not expected to be cognitively demanding—specifically, stepping over a barrier—with their performance in completely different tasks that were expected to be cognitively demanding. Future work could attempt to assess how dependent the effect of specific forms of lateralization are on task performance by collecting multiple measures of laterality (e.g., eye use, whole body turns) and conducting a wider range of cognitive assays that involve the use of other parts of the body while searching for relationships between tasks relying on particular body parts and their symmetry. For example, the strength of lateralization in the use of the pheasants’ laterally placed eyes may explain accuracy in visual categorization, as is reported in domestic chicks (Rogers & Anson, 1979), pigeons (Güntürkün & Kesch, 1987), zebra finches (*Taeniopygia guttata*; Alonso, 1998), and quail (Valenti, Anna Sovrano, Zucca, & Vallortigara, 2003). Thirdly, although we presented tasks that we assumed to be distinct from one another (colour discrimination tasks and tasks used to assess spatial ability), these tests may be using similar cognitive processes such as a basic ability to form associations. The relationship between lateralization and performance is expected to be task dependent (Boles et al., 2008)—therefore, it may be revealing to measure a greater variety of cognitive processes such as inhibitory control and active working memory and try to relate them to the extent of lateralization. Fourth, and finally, we may not be conducting testing in an environment that reveals differences in cognitive performances dependent on lateralization. To obtain an accurate measure of cognitive performance, we trained the pheasants to voluntarily isolate themselves in a testing chamber in a stress-free environment, free from competition, predation risk, and social confounds. However, benefits of lateralization on cognitive performance may only be evident when the animal is presented with multiple challenges. For instance, when predators were absent, teleost fish (*Giradinus falcatus*) did not differ in the rate they were able to catch prey, but when in the presence of a predator, individuals with greater lateralization were quicker compared with nonlateralized fish (Dadda & Bisazza, 2006). Domestic chicks that were more lateralized were better at discriminating food from nonfood when in the presence of a predator compared to less lateralized individuals (Rogers et al., 2004). In order to find an effect of lateralization on cognitive performance we may need to present pheasants with cognitive tasks in conjunction with additional stimuli, such as in the presence of conspecifics (to induce competition) or in the presence of a predator (playback or dummy). If this is the case, then the fitness benefits of lateralization on cognitive performance may only be revealed in the wild where animals must exact behaviours when subject to natural stressors.

Our finding that individuals with stronger footedness do not have especially good or poor cognitive performance suggests that associated differences in cognitive ability cannot explain our previous findings that individuals with moderate levels of footedness survived in the wild better (Whiteside et al., 2018), nor can it explain what may be constraining extreme levels of footedness in pheasants whilst maintaining substantial individual variation. It could be that pheasants with greater degrees of lateralization behave in a way that makes them more susceptible to predation. This may be a direct effect because pheasants are gregarious (Ridley & Hill, 1987; Whiteside et al., 2018), and if a high degree of lateralization generates predictable movement patterns when attacked, such individuals expressing these movements may be targeted by predators (Vallortigara & Rogers, 2005). The effect may also be indirect. Strongly lateralized convict cichlids (*Amatitlania nigrofasciata*) are quicker to emerge from a refuge indicative of boldness (Reddon & Hurd, 2009), which may expose them to predators and so influence survival (Smith & Blumstein, 2008). The degree of laterality may also come at the cost of being more sensitive to stressors, as seen in Port Jackson sharks (*Heterodontus portusjacksoni*; Byrnes, Vila Pouca, & Brown, 2016). At present, it remains unclear why strongly footed pheasants are more likely to die, but our current work suggests that it is not because this exaggerated lateralization is accompanied by particularly good or poor expression of basic cognitive abilities.

Very few animal populations are uniformly and extremely lateralized. This is perhaps surprising because we might expect strong directional selection on lateralization, with much previous research suggesting that lateralized individuals benefit because of enhanced cognitive ability (for a review, see Rogers et al., 2013), as well as in other fitness enhancing behaviours—for example, foraging efficiency (Kurvers et al., 2017; McGrew & Marchant, 1999; Rogers, 2000; Rogers et al., 2004), migration (Found & St. Clair, 2017), detecting (model) predators (Rogers, 2000), courtship (Krakauer et al., 2016), and intrasexual contests (Jennings, 2012). Our null results support the growing evidence that greater levels of lateralization are not necessarily beneficial for an individual’s cognitive ability (Brown & Braithwaite, 2004; Dadda et al., 2009), and this reduces or removes one opportunity for selection to act on this trait.

## Electronic supplementary material

ESM 1 (XLSX 24 kb)

## References

[CR1] Alonso Y (1998). Lateralization of visual guided behaviour during feeding in zebra finches (*Taeniopygia guttata*). Behavioural Processes.

[CR2] Andrew R, Mench J, Rainey C, Ingle DJ, Goodale MA, Mansfield RJ (1982). Right–left asymmetry of response to visual stimuli in the domestic chick. *Analysis of visual behaviour*.

[CR3] Bell, A. T., & Niven, J. E. (2016). Strength of forelimb lateralization predicts motor errors in an insect. *Biology Letters, 12.*10.1098/rsbl.2016.0547 10.1098/rsbl.2016.0547 PMC5046935 27651534

[CR4] Boles DB, Barth JM, Merrill EC (2008). Asymmetry and performance: Toward a neurodevelopmental theory. Brain and Cognition.

[CR5] Brown C, Braithwaite VA (2004). Effects of predation pressure on the cognitive ability of the poeciliid *Brachyraphis episcopi*. Behavioral Ecology.

[CR6] Byrnes EE, Vila Pouca C, Brown C (2016). Laterality strength is linked to stress reactivity in Port Jackson sharks (*Heterodontus portusjacksoni*). Behavioural Brain Research.

[CR7] Cantalupo C, Vila Pouca C, Brow G (1995). Lateralization of predator-evasion response in a teleost fish (*Girardinus falcatus*). Neuropsychologia.

[CR8] Casey MB (2005). Asymmetrical hatching behaviors: The development of postnatal motor laterality in three precocial bird species. Developmental Psychobiology.

[CR9] Corballis MC, Hofman MA, Falk D (2012). Lateralization of the human brain. *Progress in brain research*.

[CR10] Dadda M, Agrillo C, Bisazza A, Brown C (2015). Laterality enhances numerical skills in the guppy, *Poecilia reticulata*. Frontiers in Behavioral Neuroscience.

[CR11] Dadda M, Bisazza A (2006). Does brain asymmetry allow efficient performance of simultaneous tasks?. Animal Behaviour.

[CR12] Dadda M, Zandona E, Agrillo C, Bisazza A (2009). The costs of hemispheric specialization in a fish. Proceedings of the Royal Society B: Biological Sciences.

[CR13] de Kloet ER, Oitzl MS, Joëls M (1999). Stress and cognition: are corticosteroids good or bad guys?. Trends in Neurosciences.

[CR14] Department for Environment, Food and Rural Affairs (2009). *Code of practice for the welfare of game birdsreared for sporting purposes*.

[CR15] De Santi A, Sovrano V, Bisazza A, Vallortigara G (2001). Mosquitofish display differential left-and right-eye use during mirror image scrutiny and predator inspection responses. Animal Behaviour.

[CR16] Dharmaretnam M, Vijitha V, Priyadharshini K, Jashini T, Vathany K (2002). Ground scratching and preferred leg use in domestic chicks: Changes in motor control in the first two weeks post-hatching. Laterality: Asymmetries of Body, Brain and Cognition.

[CR17] Diekamp B, Prior H, Güntürkün O (1999). Functional lateralization, interhemispheric transfer and position bias in serial reversal learning in pigeons (*Columba livia*). Animal Cognition.

[CR18] Found, R., & St. Clair, C. C. (2017). Ambidextrous ungulates have more flexible behaviour, bolder personalities and migrate less. *Royal Society Open Science, 4*(2)*.*10.1098/rsos.160958 10.1098/rsos.160958 PMC5367311 28386447

[CR19] Frasnelli E, Iakovlev I, Reznikova Z (2012). Asymmetry in antennal contacts during trophallaxis in ants. Behavioural Brain Research.

[CR20] Frasnelli E, Vallortigara G, Rogers LJ (2012). Left–right asymmetries of behaviour and nervous system in invertebrates. Neuroscience & Biobehavioral Reviews.

[CR21] Güntürkün O, Diekamp B, Manns M, Nottelmann F, Prior H, Schwarz A, Skiba M (2000). Asymmetry pays: Visual lateralization improves discrimination success in pigeons. Current Biology.

[CR22] Güntürkün O, Kesch S (1987). Visual lateralization during feeding in pigeons. Behavioral neuroscience.

[CR23] Hill D, Robertson P (1988). *The pheasant: Ecology, management and conservation*.

[CR24] Hirnstein M, Leask S, Rose J, Hausmann M (2010). Disentangling the relationship between hemispheric asymmetry and cognitive performance. Brain and Cognition.

[CR25] Jennings DJ (2012). Right-sided bias in fallow deer terminating parallel walks: Evidence for lateralization during a lateral display. Animal Behaviour.

[CR26] Krakauer AH, Blundell MA, Scanlan TN, Wechsler MS, McCloskey EA, Yu JH, Patricelli GL (2016). Successfully mating male sage-grouse show greater laterality in courtship and aggressive interactions. Animal Behaviour.

[CR27] Kurvers RHJM, Krause S, Viblanc PE, Herbert-Read JE, Zaslansky P, Domenici P, Krause J (2017). The evolution of lateralization in group hunting sailfish. Current Biology.

[CR28] LaMendola NP, Bever TG (1997). Peripheral and cerebral asymmetries in the rat. Science.

[CR29] Langley EJ, van Horik JO, Whiteside MA, Beardsworth CE, Madden JR (2018). The relationship between social rank and spatial learning in pheasants, *Phasianus colchicus*: Cause or consequence?. PeerJ.

[CR30] Levy J (1969). Possible basis for the evolution of lateral specialization of the human brain. Nature.

[CR31] Madden JR, Langley EJ, Whiteside MA, Beardsworth CE, van Horik JO (2018). The quick are the dead: Pheasants that are slow to reverse a learned association survive for longer in the wild. Philosophical Transactions of the Royal Society B: Biological Scien es.

[CR32] Magat M, Brown C (2009). Laterality enhances cognition in Australian parrots. Proceedings of the Royal Society B: Biological Sciences.

[CR33] McGrew WC, Marchant LF (1999). Laterality of hand use pays off in foraging success for wild chimpanzees. Primates.

[CR34] McManus I (2002). *Right hand, left hand*.

[CR35] Mendl M (1999). Performing under pressure: Stress and cognitive function. Applied Animal Behaviour Science.

[CR36] Miler K, Kuszewska K, Woyciechowski M (2017). Larval antlions with more pronounced behavioural asymmetry show enhanced cognitive skills. Biology Letters.

[CR37] Ocklenburg S, Güntürkün O (2017). *The lateralized brain: The neuroscience and evolution of hemispheric asymmetries*.

[CR38] Pascual A, Huang K-L, Neveu J, Préat T (2004). Neuroanatomy: Brain asymmetry and long-term memory. Nature.

[CR39] R Development Core Team (2014). R: A language and environment for statistical computing [Computer software].

[CR40] Reddon AR, Hurd PL (2009). Individual differences in cerebral lateralization are associated with shy–bold variation in the convict cichlid. Animal Behaviour.

[CR41] Ridley M, Hill D (1987). Social organization in the pheasant (*Phasianus colchicus*): Harem formation, mate selection and the role of mate guarding. Journal of Zoology.

[CR42] Rogers LJ (2000). Evolution of hemispheric specialization: Advantages and disadvantages. Brain and Language.

[CR43] Rogers, L. J. (2002). Lateralization in vertebrates: its early evolution, general pattern, and development. In P. J. B. Slater, J. S. Rosenblatt, C. T. Snowdon, & T. J. Roper (Eds.), *Advances in the study of behavior* (Vol. 31, pp. 107–161). 10.1016/S0065-3454(02)80007-9

[CR44] Rogers LJ, Anson J (1979). Lateralisation of function in the chicken fore-brain. Pharmacology Biochemistry and Behavior.

[CR45] Rogers LJ, Vallortigara G, Andrew RJ (2013). *Divided brains: The biology and behaviour of brain asymmetries*.

[CR46] Rogers LJ, Zucca P, Vallortigara G (2004). Advantages of having a lateralized brain. Proceedings of the Royal Society of London Series B: Biological Sciences.

[CR47] Smith BR, Blumstein DT (2008). Fitness consequences of personality: A meta-analysis. Behavioral Ecology.

[CR48] Ströckens F, Güntürkün O, Ocklenburg S (2013). Limb preferences in non-human vertebrates. Laterality: Asymmetries of Body, Brain and Cognition.

[CR49] Tommasi L, Gagliardo A, Andrew RJ, Vallortigara G (2003). Separate processing mechanisms for encoding of geometric and landmark information in the avian hippocampus. European Journal of Neuroscience.

[CR50] Tommasi L, Vallortigara G (1999). Footedness in binocular and monocular chicks. Laterality: Asymmetries of Body, Brain and Cognition.

[CR51] Valenti A, Anna Sovrano V, Zucca P, Vallortigara G (2003). Visual lateralisation in quails (*Coturnix coturnix*). Laterality: Asymmetries of Body, Brain and Cognition.

[CR52] Vallortigara G, Rogers LJ, Kaplan G (2004). Visual cognition and representation in birds and primates. *Comparative vertebrate cognition*.

[CR53] Vallortigara G, Pagni P, Sovrano VA (2004). Separate geometric and non-geometric modules for spatial reorientation: Evidence from a lopsided animal brain. Journal of Cognitive Neuroscience.

[CR54] Vallortigara G, Rogers LJ (2005). Survival with an asymmetrical brain: Advantages and disadvantages of cerebral lateralization. Behavioral and Brain Sciences.

[CR55] van Horik JO, Langley EJ, Whiteside MA, Laker PR, Madden JR (2018). Intra-individual variation in performance on novel variants of similar tasks influences single factor explanations of general cognitive processes. Royal Society Open Science.

[CR56] van Horik JO, Langley EJ, Whiteside MA, Madden JR (2018). A single factor explanation for associative learning performance on colour discrimination problems in common pheasants (*Phasianus colchicus*). Intelligence.

[CR57] Versace E, Vallortigara G (2015). Forelimb preferences in human beings and other species: Multiple models for testing hypotheses on lateralization. Frontiers in Psychology.

[CR58] Whiteside MA, Bess MM, Frasnelli E, Beardsworth CE, Langley EJ, van Horik JO, Madden JR (2018). Low survival of strongly footed pheasants may explain constraints on lateralization. Scientific Reports.

[CR59] Whiteside MA, Sage R, Madden JR (2016). Multiple behavioural, morphological and cognitive developmental changes arise from a single alteration to early life spatial environment, resulting in fitness consequences for released pheasants. Royal Society Open Science.

[CR60] Whiteside MA, van Horik JO, Langley EJG, Beardsworth CE, Capstick LA, Madden JR (2018). Patterns of association at feeder stations for common pheasants released into the wild: Sexual segregation by space and time. Ibis.

